# Wearables research for continuous monitoring of patient outcomes: A scoping review

**DOI:** 10.1371/journal.pdig.0000860

**Published:** 2025-05-09

**Authors:** Kalee Lodewyk, Madeleine Wiebe, Liz Dennett, Jake Larsson, Andrew Greenshaw, Jake Hayward

**Affiliations:** 1 Department of Psychiatry, Faculty of Medicine and Dentistry, University of Alberta, Edmonton, Alberta, Canada; 2 Faculty of Medicine and Dentistry, University of Alberta, Edmonton, Alberta, Canada; 3 Geoffrey and Robyn Sperber Health Sciences Library, University of Alberta, Edmonton, Alberta, Canada; 4 Department of Emergency Medicine, Faculty of Medicine and Dentistry, University of Alberta, Edmonton, Alberta, Canada; Iran University of Medical Sciences, IRAN, ISLAMIC REPUBLIC OF

## Abstract

**Background:**

The use of wearable devices for remote health monitoring is a rapidly expanding field. These devices might benefit patients and providers; however, they are not yet widely used in healthcare. This scoping review assesses the current state of the literature on wearable devices for remote health monitoring in non-hospital settings.

**Methods:**

CINAHL, Scopus, Embase and MEDLINE were searched until August 5, 2024. We performed citation searching and searched Google Scholar. Studies on wearable devices in an outpatient setting with a clinically relevant, measurable outcome were included and were categorized according to intended use of data: monitoring of existing disease vs. diagnosis of new disease.

**Results:**

Eighty studies met eligibility criteria. Most studies used device data to monitor a chronic disease (68/80, 85%), most often neurodegenerative (22/68, 32%). Twelve studies (12/80, 15%) used device data to diagnose new disease, majority being cardiovascular (9/12, 75%). A range of wearable devices were studied with watches and bracelets being most common (50/80, 63%). Only six studies (8%) were randomized controlled trials, four of which (67%) showed evidence of positive clinical impact. Feasibility determinants were inconsistently reported, including compliance (51/80, 64%), patient-reported useability (13/80, 16%), and participant technology literacy (1/80, 1%).

**Conclusions:**

Evidence for clinical effectiveness of wearable devices remains scant. Heterogeneity across studies in terms of devices, disease targets and monitoring protocols makes data synthesis challenging, especially given the rapid pace of technical innovation. These findings provide direction for future research and implementation of wearable devices in healthcare.

## Author summary

Wearable devices, such as smartwatches, have potential to benefit patients and assist healthcare workers; however, these devices are not yet widely used in healthcare. This review looks at existing research around wearable devices used to monitor health markers outside of the hospital. We worked with a research librarian to comprehensively search online databases until August 5, 2024. Studies on use of wearable devices outside of a hospital setting with clinically relevant, measurable outcomes were included and sorted by whether they used data to monitor an existing disease or identify a new disease. We included 80 studies: most focused on monitoring existing chronic diseases (68/80, 85%), while the minority looked at identifying a new disease (12/80, 15%). Many different wearable devices were investigated in these studies. A small proportion (6/80, 8%) of the studies were randomized controlled trials. Four of these six studies (67%) showed a positive clinical impact. We also found that feasibility information was reported inconsistently. There is limited evidence for the clinical effectiveness of wearable devices. Studies varied on device used, disease target, and monitoring protocol, making it difficult to draw conclusions based on this literature, especially since the technology is rapidly evolving.

## Introduction

The global market for wearable electronics was estimated at US$32.5 billion in 2022 and is projected to reach US$173.7 billion by 2030, growing at an annual rate of 23.3% over this period [1]. These devices can measure an expanding array of biometrics, including heart rate, blood pressure, and oxygen saturation, and more [2]. They are increasingly integrated with daily life, offering new ways to monitor health unobtrusively for large, distributed populations [2–5]. Remote health monitoring (RHM) is rapidly gaining interest in healthcare research, leveraging wearable technologies, like smartwatches, to track health status in non-hospital environments [3,6]. In some contexts, RHM has been shown to shorten hospital stays [7], reduce hospital readmissions [8], lower healthcare costs [9], and decrease clinician burnout [10].

Despite immense potential, real-world implementations of RHM with wearable devices remain limited. Most published use-cases are for the most common, chronic diseases like diabetes, hypertension, chronic obstructive pulmonary disorder (COPD) or congestive heart failure (CHF), and evidence for effectiveness is conflicting [6]. Further, available data are derived from a diverse set of devices, many of which are now out of date, in a range of patient populations, using different biometric parameters and disease outcomes [3]. Researchers are increasingly exploring wearable devices to improve health outcomes across a broad range of disease contexts. While recent reviews have examined clinical applications of wearables, they primarily focus on chronic disease [11,12]. Given the rapid evolution of wearable technologies and the growing number of potential use cases, there is a need to update existing reviews to include non-chronic conditions and provide a more comprehensive overview of their potential impact.

We therefore performed a scoping review exploring the current clinical applications for wearable devices and RHM for non-hospital settings, summarizing evidence specifically on the impact on patient outcomes, investigating for actual change in these outcomes due to use of a wearable device.

## Methods

The protocol for this scoping review was published on Open Science Framework (https://doi.org/10.17605/OSF.IO/AT7VS). We followed Arksey and O’Malley’s guidelines for scoping review methods [13].

### Eligibility criteria

The inclusion and exclusion criteria for this scoping review (Table 1) were developed around the PCC (population, concept, context) framework, as per the Joanna Briggs Institute for scoping reviews [14]. We targeted an adult population, excluding studies including patients under 18 years of age. For inclusion, studies needed to use a wearable device to monitor disease. Therefore, studies including solely healthy subjects were excluded. Wearable devices had to meet the following definition based on Gao et al. 2016: devices that can be worn on human skin to continuously and closely monitor an individual’s activities, without interrupting or limiting the user’s motions” [5]. Examples include smartwatches and wearable textiles/clothing with embedded sensors.

**Table 1:** Study eligibility criteria, based on the PCC framework (Population, Concept, Context), as recommended by the Joanna Briggs Institute for Scoping Reviews [14].

	Inclusion criteria	Exclusion criteria
**Population**	• Adults (18 years of age and above)	• Children (under 18 years of age)
**Concept**	• Wearable devices (according to a commonly used definition (5)) for remote health monitoring • Disease monitoring (changes in an existing disease or detection of a new disease) • Clinically relevant, measurable outcome	• Any technology that does not meet the definition for a wearable device • Healthy subjects
**Context**	• Outpatient monitoring	• Inpatient monitoring
**Study design**	• Randomized controlled trials • Longitudinal studies • Observational studies	• Conference proceedings • Abstracts • Validation studies • Opinion pieces • Cross-sectional studies
**Language**	• Studies written in the English language	• Studies that were not written in the English language

We focused on outpatient or non-hospital RHM, excluding studies with hospitalized populations and long-term care facilities. This was done for two main reasons: 1) our primary interest was in settings where clinical teams are less able to support patients in their use of technology for more accurate data on usability and acceptability; and 2) to increase feasibility of the search given an expansive literature base. We also excluded studies with implantable devices, such as blood glucose monitors because they do not meet our definition for ‘wearable’ [5]. All trial designs were eligible, including randomized controlled trials, longitudinal studies, feasibility studies, and observational studies. We focused on studies that measured a clinical outcome, and therefore validation studies were excluded. Conference proceedings, opinion pieces, reviews, and cross-sectional studies were also excluded. Only studies written in the English language were included as we did not have the resources to support a translation service.

### Search strategy and study selection

A research librarian (coauthor LD) assisted in designing our search strategy. We searched the following electronic databases from inception to August 5, 2024: CINAHL (1937-present), Scopus (2004-present), Embase (1974-present) and MEDLINE (1946-present via OVID). Our search strategy for MEDLINE is shown in S1 File. This search strategy was translated for use in the other databases. We searched Google Scholar on January 23, 2024. Covidence Systematic Review software [15] was used for study screening. Two reviewers (KL, MW) independently screened studies at both the title/abstract and full text stages. A third reviewer (JH) was involved to resolve conflicts. Citation searching was performed for included studies.

### Data extraction and analysis

A data extraction spreadsheet was developed based on the review question and PCC. Two reviewers (KL, MW) completed data extraction. Extracted data included study characteristics (e.g., study design, mean participant age, country), wearable device information (device name, company, sensor type, body part, length of monitoring, instructions on use, frequency of removal, and technical support, and parameters monitored), control group (where applicable), and study findings (including clinical and economic outcomes, protocol compliance, device useability and feasibility).

## Results

### Search results and study selection

**Fig 1:**
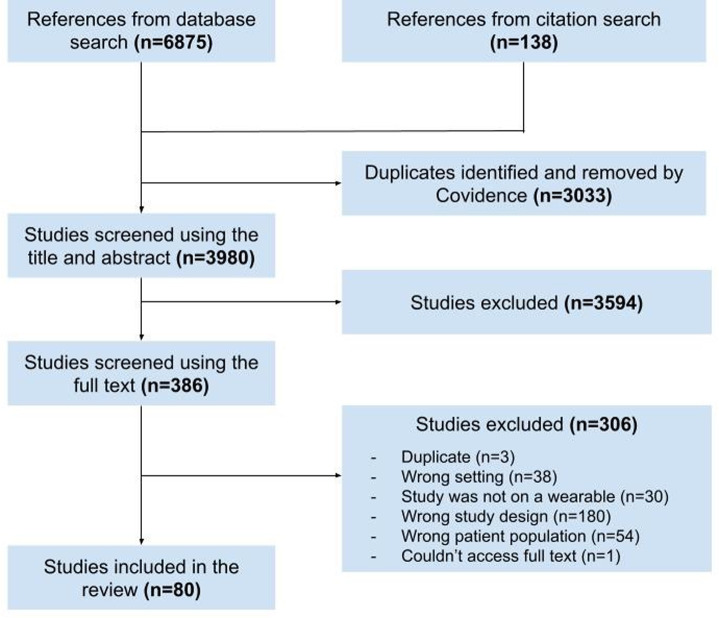
PRISMA flow diagram outlining the study selection process, including reasons for exclusion at the full text review stage.

Fig 1 shows the PRISMA flow diagram summarizing search results and study selection. Our search identified 3980 studies after the removal of duplicates. Title and abstract screening resulted narrowed this to 386 studies for full-text screening; the final sample included 80 studies that met our eligibility criteria.

### Study characteristics

Table 2 provides a summary of key study characteristics of the included studies, categorized by disease category (i.e., body system) and outcome type (i.e., monitoring of existing disease or diagnosis of new disease). The oldest study was published in 2001, with the majority of included studies published between 2019 and 2024 (51 studies, 64%). The most frequent study location was the United States of America (40 studies, 50%), followed by the UK (10 studies, 13%) and the Netherlands (8 studies, 10%). Only three studies (4%) reported including rural patients. Six of the included studies were randomized controlled trials (RCTs) (8%). Control groups were present in 22 studies (28%).

**Table 2:** Summary of main study characteristics for each included study (n = 80).

Monitoring disease										
Study		Country	Study design	Control	Sample size	Age	Disorder/ monitored parameter(s)	Monitoring length	Wearable device name	Summary of main finding(s) related to clinical outcomes
**Cardiovascular disease**										
[16]	Garcia et al., 2023	France	Prospective, Cohort	No	1013	61	Heart failure/ nocturnal heart rate	Median 68 days	LifeVest, external defibrillator	High nocturnal heart rate was significantly associated with the primary endpoint (a composite of cardiovascular death and heart failure-related hospitalization).
[17]	Kim et al., 2020	Korea	Prospective, non- randomized, interventional	No	110	N/R	Stroke/ blood pressure, physical activity	12 weeks	Bluetooth sphygmomanometer, wrist-worn smart band	26 patients with uncontrolled blood pressure experienced a decrease in blood pressure.
[18]	Kolk et al., 2023	Netherlands, Denmark	Prospective, observational	No	303	62.9	Ischaemic cardiomyopathy, heart failure/ activity, sleep, step count	6 months	GENEActiv accelerometer	Digital biomarkers showed significant correlations to patient-reported physical and social limitations, severity and frequency of symptoms and quality of life.
[19]	Okumus et al., 2018	Turkey	Observational	No	38	50	Pulmonary hypertension/ daily activity and energy expenditure	N/R	Senseband	Significant correlations between items of the Nottingham extended activity of daily living index and activity monitor data.
[20]	Pothineni et al., 2022	USA	Cohort	No	20	64	Stroke/ ECG	2 weeks	ZIO XT patch	Arrhythmias were observed in 2/3 of patients recovering from stroke.
[21]	Rosenberg et al., 2013	USA	Comparative study	No	74	65	Atrial fibrillation/ ECG	2 weeks	Zio XT Patch	All atrial fibrillation episodes were recorded on the Holter and the wearable. The wearable detected significantly more AF episodes than the Holter due to a longer monitoring time. The wearable also detected other arrhythmias.
[22]	Stehlik et al., 2020	USA	Observational	No	100	68	Heart failure/ ECG, temperature, accelerometry data, skin impedance	3 months	Vital Connect	Combination of device parameters detected precursors of hospitalization for heart failure exacerbation with high sensitivity and specificity.
[23]	Stollfuss et al., 2021	Germany	Prospective, cohort, observational	No	31	N/R	Pulmonary arterial hypertension/ steps, distance walked, heart rate, motion	3 months	Apple Watch Series 2	Daily physical activity increased during the study and so did 6-minute walk distance.
[24]	Tung et al., 2014	USA	Observational	No	N/R	68	Stroke, transient ischemic attack/ ECG	2 weeks	ZIO patch	The wearable device accurately captured atrial fibrillation burden in patients recovering from a stroke.
**Diabetes**										
[25]	Abbott et al., 2019	UK	Prospective, RCT	Yes	58	59*	Diabetic foot ulcers/ plantar pressure	N/R	SurroSense Rx	The intervention group received alerts and offloading instructions, leading to a 71% reduction in ulcer incidence.
[26]	Armstrong et al., 2004	USA	Prospective, longitudinal	No	100	69	Diabetic foot ulcers/ steps taken over a period of time	25 weeks	Biotrainer Pro	Average daily activity was significantly lower in participants who ulcerated compared to those who did not ulcerate. No significant difference in average daily activity during that period.
[27]	Najafi et al., 2017	USA	Prospective, cohort	No	17	62	Diabetic foot ulcers/ plantar pressure	3 months	SurroSense Rx	Participants who received at least 1 alert every 2 hours were more adherent with offloading and successfully offloaded faster than those who received fewer alerts.
[28]	Reyzelman et al., 2018	USA	Case studies, observational	No	34	62	Diabetic foot ulcers/ foot temperature	1 week	Siren Diabetic Socks	Temperature measurements from the socks were consistent with initial observations and medical history.
**Cancer**										
[29]	Dadhania et al., 2023	UK	Cohort	Yes	42	N/R	High grade glioma/ accelerometer	Median 49 days	Axivity AX3 triaxial accelerometer	Mean acceleration and daily time spent walking correlated positively with global health quality of life and physical functioning scores and inversely with the fatigue score.
[30]	Gresham et al., 2018	USA	Prospective, single cohort	No	37	N/R	Cancer (stage 3 or 4 pancreatic, colorectal, other gastrointestinal, gynecological, or lung cancer)/ steps, distance, flights of stairs	2 weeks	Fitbit Charge HR	Correlations were observed between average daily steps and measures of patient functioning and ability of self-care. Increases of 1000 steps were associated with reduced odds for adverse events, hospitalizations, and hazard for death.
[31]	Korde et al., 2023	USA	Prospective, observational, cohort	No	40	41-82	Multiple myeloma/ continuous activity	6 months	Garmin Vivofit	Overall activity trended upward cycle over cycle for the entire study cohort. Activity trends associated with improvement of electronic patient reported outcome domains, including physical functioning scores, global health scores, and declining disease burden symptom scores.
[32]	Low et al., 2017	USA	Exploratory	No	14	60	Gastrointestinal cancer/ activity and sleep metrics	4 weeks	Fitbit Charge HR	Increased symptom burden was associated with reduced mobility and sleep (more sleep and increased periods of waking).
[32]	Low et al., 2021	USA	Prospective, longitudinal	No	44	66	Surgery for pancreatic cancer/ step count, distance traveled	12 weeks	Fitbit Charge 2	Predicted next-day diarrhea, fatigue and pain with high accuracy.
[33]	Low et al., 2024	USA	Prospective, longitudinal	No	40	73.35	Any cancer/ activity metrics, time spent at home	Mean 26 days	Fitbit inspire 3	More walking, faster speeds and smoother movement were linked to better physical function. After adjusting for age and health conditions, these device parameters still showed a connection to physical function, and peak walking speed was associated with fall risk.
[34]	Ohri et al., 2019	USA	Prospective	No	50	N/R	Locally advanced non-small cell lung cancer	N/R	Garmin Vivofit	Inactive participants were more likely to be hospitalized and less likely to complete radiation therapy without delay exceeding 1 week.
**Renal disease**										
[35]	Cohen et al., 2022	USA	Pilot	No	48	60	End stage kidney disease/ step count	6 months	Garmin Vivofit 2	Step count was lower on dialysis days. Step count was higher in the hour following dialysis, and these increases were greater in patients with a fast recovery time or a high short physical performance battery score.
[36]	Lunney et al., 2021	Canada	Prospective observational cohort feasibility study	No	46	N/R	Kidney failure/ daily step count	4 weeks	Fitbit Alta	No association between step count and the occurrence of clinical events, although the number of events was very small.
**Respiratory diseases**										
[37]	Drent et al., 2020	Netherlands	Prospective, cohort	Yes	149	N/R	Sarcoidosis/ Number of steps, estimated distance, number of flights climbed, activity minutes	12 weeks	Fitbit Charge HR	Exercise capacity increased to a greater extent in the physical activity group compared to the control group..
[38]	Hawthorne et al., 2022	UK	Prospective, cohort, observational	No	31	N/R	COPD/ respiratory rate, heart rate, skin temperature, physical activity	6 weeks	Equivital EQ02 + LifeMonitor	Increased heart rate and reduced physical activity were associated with worsening symptoms.
[39]	Orme et al., 2018	UK	RCT	No	33	N/R	COPD/ sitting time, stand-ups, steps	2 weeks	N/R	Step count increased, but stationary time and physical activity did not. No statistically significant change in COPD symptoms, fatigue, anxiety or fear of falling. Severe fatigue decreased.
[40]	Pedone et al., 2013	Italy	RCT	Yes	99	74	COPD/ heart rate, physical activity, near-body temperature, galvanic skin response	9 months	SweetAge monitoring system wristband, pulse oximeter	Incidence rate of respiratory events was lower in the telemonitoring group.
[41]	Rubio et al., 2017	UK	Observational	No	18	72	COPD/ breathing rate	6 weeks	Chest-band	A reduction in resting breathing rate during the recovery from an acute exacerbation of COPD was observed in some but not all participants and there was considerable day-to-day individual variation.
[42]	Tabak et al., 2014	Netherlands	Pilot RCT	Yes	30	65*	COPD/ steps per day	4 weeks	MTx-W sensor	Wearable device data provided alerts around physical activity levels. There was a non-significant difference in improvement in health status between the monitored and usual care groups.
[43]	Wu et al., 2021	Taiwan	Prospective, cohort	No	67	67	COPD/ walking steps, climbing stairs, distances, consumption in calories, heart rate, sleep status	4 months	Fitbit Versa	Device data predicted acute exacerbation of COPD by 7-days (92.1% accuracy). The most important variables in the model were daily steps walked, stairs climbed, and daily distance moved.
[44]	Wurzer et al., 2021	Germany	Feasibility	No	153	–	COVID-19/ oxygen saturation, respiratory rate, heart rate, temperature	9 days median	Cosinuss One in-ear sensor	There was a lower SpO2 before hospitalization than for participants who were not hospitalized.
[45]	Yamagami et al., 2021	Japan	Prospective	No	23	51	COVID-19/ resting heart rate, sleep quality, steps, physical activity, sleep quality, estimated oxygen variation	30 days	Fitbit Charge 3	Oxygen saturation on device correlated with COVID-19 symptom severity.
**Musculoskeletal disorders**										
[46]	Gurchiek et al., 2019	USA	Observational	Yes	28	26*	Anterior cruciate ligament surgery/ gait, daily step count	17 weeks	BioStamp	Asymmetry indices (measured with the wearable device) were more strongly correlated with recovery time than standard step counts. Asymmetry indices were significantly different between patients and controls.
[47]	Maharaj et al., 2022	Australia	Prospective, observational	No	50	24	Spine surgery for lumbar spinal stenosis or degenerative spondylolisthesis/ step count, distance traveled	12 weeks	Mi Band 2 smartwatch	Mean daily step count and daily walking distance significantly improved over postoperative course.
[48]	Patterson et al., 2020	USA	Prospective, cohort, observational	No	20	63	Total joint arthroplasty (hip, knee)/ steps, distance, floors climbed, calories expended, active minutes	8 weeks	Fitbit Flex	Decreased activity group reported greater clinically relevant pain reduction.
[49]	Perraudin et al., 2018	Ireland	Observational	Yes	45	N/R	Arthritis/ movement	4 weeks	Actigraph GT9X Link	Five times sit to stand test performance (time to complete) was associated with pain and stiffness intensity.
[50]	Scheer et al., 2017	USA	Prospective, cohort	No	32	58	Surgery for spinal deformity or degenerative disease/ number of daily steps, max hourly steps, activity intensity	6 months	Fitbit Flex	Health-related quality of life measures were correlated with preoperative average daily steps, but not with post-operative steps.
[51]	Toogood et al., 2016	USA	Prospective, cohort	No	33	71	Total hip arthroplasty/ mean daily step count	30 days	Fitbit	Number of steps correlated with recovery time. Age and an anterior surgical approach were associated with increased activity.
**Neurodegenerative disorders**										
[52]	Block et al., 2016	USA	Prospective, cohort, observational, longitudinal	No	80	50	Multiple sclerosis/ step count	4 weeks	Fitbit Flex	Greater MS disability associated with lower daily step count.
					50	48		6 months		Greater MS disability associated with lower average daily step count.
[53]	Block et al., 2017	USA	Prospective, cohort, RCT	Yes	99	50*	Multiple sclerosis/ step count	4 weeks	Fitbit Flex 2	Negative correlation between disability level and step count.
[54]	Block et al., 2019	USA	Prospective, cohort, observational, longitudinal	No	95	50	Multiple sclerosis/ step count	1 year	Fitbit Flex	Greater MS disability associated with lower average daily step count.
[55]	Block et al., 2021	USA	RCT	Yes	21	48*	Multiple sclerosis/ step count	12 weeks	Fitbit Flex	Monitoring led to better adherence to clinical recommendations.
[56]	Burq et al., 2021	Netherlands	Prospective, longitudinal	No	388	63	Parkinson’s disease/ motor signs	1 year	Verily Study Watch 2nd Generation	Smartwatch measurements were correlated with rest tremor, bradykinesia, and gait.
[57]	Caballol et al., 2023	Spain	Prospective, observational	Yes	39	69	Parkinson’s disease/ movement	1 week before treatment, 1 week 3 months after therapeutic changes	STAT-ONTM system	Device data correlated with on-time, off-time, number of steps, gait fluidity, motor fluctuations, dyskinesia, and freezing of gait, in agreement with clinical measures.
[58]	Cereda et al., 2010	Italy	Prospective, crossover	Yes	6	N/R	Parkinson’s disease/ total energy expenditure, physical activity, number of steps, metabolic rate	2 weeks	SenseWear Armband	Wearable measurements correlated with dyskinesias; no correlation with number of steps for participants who ate a low-protein diet.
[59]	Dalla Costa et al., 2024	Italy, Spain, Denmark	Observational	No	306	45.6	Multiple Sclerosis/ daily steps	2 years	Fitbit charge 3	Daily steps recorded by the Fitbit is inversely correlated with EDSS (disability) score.
[60]	Davies et al., 2020	USA	Prospective, observational	No	8	44	Tay-Sachs and Sandhoff disease/ average daily maximum, average daily steps, average daily steps per 30-minute epoch	6 months	Wristwatch	Negative correlation between disease impact/severity and wearable metrics.
[61]	Fay-Karmon et al., 2024	N/R	Observational	No	21	66.1	Parkinson’s disease/ tremor, dyskinesia, level of activity	2 weeks	Apple watch series 4	Differences among individual symptoms’, including levodopa-related variations, led to grouping of participants into four data-driven profiles.
[62]	Gordon et al., 2019	N/R	Observational	No	15	N/R	Huntington’s disease/ movement	6 months	Smartwatch	Correlation between device data and patient-reported chorea score. Model including device data predicted chorea severity.
[63]	Joshi et al., 2019	USA	Observational	No	63	N/R	Parkinson’s disease/ movement	6 days	Personal KinetiGraph system	The wearable device detected bradykinesia or dyskinesia despite patients not reporting these symptoms and improved patient-provider communication, ability to assess treatment impact and motor assessments.
[64]	Klassen et al., 2007	Canada	Exploratory, descriptive	Yes	39	N/R	Multiple sclerosis/ accelerometer data	4 days	TriTrac RT3 accelerometer	Accelerometer scores were significantly correlated with neurological status and disease severity.
[65]	Kotschet et al., 2022	Australia	Feasibility	No	25	60	Parkinson’s disease/ movement	6 days	Parkinson’s KinetiGraph system	Correlations observed between wearable data and chorea and bradykinesia scores.
[66]	Kratz et al., 2019	USA	Observational	No	107	N/R	Multiple Sclerosis/ physical activity	7 days	PRO-Diary	Increased fatigue and depressed mood were associated with reduced physical activity. Physical activity was not associated with pain or cognitive symptoms.
[67]	Mancini et al., 2015	USA	Feasibility	Yes	32	65*	Parkinson’s disease/ turning (velocity, steps)	1 week	Opal inertial sensors	Turn velocity and number of steps per turn were correlated with symptom rating scale (motor score).
[68]	Motl et al., 2011	USA	Observational	No	292	N/R	Multiple sclerosis/ physical activity	1 week, 1 week after 6 months	ActiGraph	Associations were observed between baseline and 6-month physical activity and disability.
[69]	Powers et al., 2021	USA	Longitudinal, cohort	Yes	396	71	Parkinson’s disease/ movement	6 months	Smartwatch	Wearable measurements were correlated with tremor severity and with expert ratings of dyskinesia.
[70]	Shammas et al., 2014	Germany	Pilot study, prospective	No	11	N/R	Multiple Sclerosis/ step count, maximum walking speed, physical activity intensity	1 year	Activity sensor	Patients with moderate disability took less steps and had slower walking speed than those with mild disability.
[71]	vanVugt et al., 2001	Netherlands	Observational	Yes	131	45*	Huntington’s disease/ daytime motor activity	5 days	Wrist-worn activity monitor	Daytime activity was decreased in patients compared to healthy controls. Hypokinesia correlated with impaired voluntary movements, disturbed gait and posture, and reduced functional capacity, progression of functional disability.
[72]	Weiss et al., 2014	Israel	Longitudinal, observational	Yes	107	N/R	Parkinson’s disease/ variability of the gait pattern, gait rhythmicity and consistency and gait smoothness	3 days	DynaPort Hybrid system	Device parameters correlated with validated measures of fall risk. Wearable data predicted time to first fall.
[73]	Woelfle et al., 2023	Switzerland	Longitudinal, observational	Yes	28	N/R	Multiple Sclerosis/ steps, activity, calories, heart rate, sleep	6 weeks	Fitbit Versa 2 smartwatch	Device parameters correlated with 3 clinical reference tests (the expanded disability status scale, timed 25-foot walk, MS-walking scale).
**Psychiatric disorders**										
[74]	Alinia et al., 2021	USA	Proof of concept	No	11	40	Stress, EtOH cravings, pain, discomfort/ ANS arousal, heart rate, heart rate variability, interbeat interval	2 weeks	Empatica E4	Electrodermal activity and heart rate variability were correlated with outcomes, including self-reported stress positive and negative emotions, and pain and discomfort.
[75]	Carreiro et al., 2020	USA	Observational	No	31	32	Substance use disorder/ electrodermal activity, skin temperature, acceleration, heart rate	4 days	E4 wearable sensor	Device parameters associated with patient-reported stress and craving..
[76]	Knight et al., 2018	Australia	Prospective, cohort, observational	No	53	21	Depression, anxiety/ daily activity duration	8 months	Fitbit, Garmin	Correlations observed between anxiety and emotional volatility scores and daily activity over 45 days of measurement.
[77]	Lahti et al., 2021	USA	Observational	No	40	40	Schizophrenia/ sleep-wake, activity count, light exposure, step count	4 months	Philips Actiwatch, Garmin Vivofit wristband	Wearable data and psychotic symptoms were correlated.
[78]	Lee et al., 2022	South Korea	Prospective, cohort	No	270	23	Major depressive disorder, bipolar disorder/ heart rate and motion	Average 280 days	Fitbit Charge HR 2 or 3	Device metrics predicted onset of depression episodes.
[79]	Mahoney et al., 2023	USA	Prospective	No	77	37.3	Substance use disorder/ heart rate, blood oxygen saturation, stress, activity, sleep	1 year	Garmin vivosmart 4	Physiological markers were significantly elevated in the week prior to drug use recurrence relative to periods of sustained abstinence.
[80]	O’Brien et al., 2017	UK	Observational	Yes	59	74*	Depression/ physical activity	7 days	Wrist-worn device	Physical activity and fine motor movement speed was associated with depression, quality of life, and associative learning.
[81]	Sun et al., 2023	UK, Spain, Netherlands	Longitudinal, observational	No	623	49	Major depressive disorder/ sleep metrics	Median 312 days	Fitbit Charge 2 or 3	Sleep onset and wakefulness correlated with PHQ-8 (patient health questionnaire for depressive symptoms)
[82]	Zhang et al., 2021	UK, Netherlands, Spain	Longitudinal, observational	No	368	N/R	Major depressive disorder/ N/R	N/R	Fitbit Charge 2 or 3	Sleep features were associated with depressive symptoms.
**Identification of new diseases**										
**Study**		**Country**	**Study design**	**Control**	**Sample size**	**Age**	**Disorder/ monitored parameter(s)**	**Monitoring length**	**Wearable device name**	**Summary of main findings**
**Cardiovascular disease**										
[83]	Ha et al., 2021	Canada	Prospective, open-label RCT	Yes	336	67	Atrial fibrillation/ ECG	30 days	SEEQ system (Medtronic), CardioSTAT system (Icentia)	Cumulative arrythmia burden was more monitored patients vs. usual care
[84]	Moshe et al., 2021	Germany	Longitudinal, observational	No	55	43	Depression, anxiety/ step count, metabolic equivalent for class, sleep	30 days	Oura ring	Sleep metrics were associated with depression. Heart rate variability was associated with anxiety. Combined data (wearable, self-reported mood and smartphone) provided the best prediction.
[85]	Pagola et al., 2023	Spain	Prospective, observational	No	224	N/R	Cryptogenic stroke/ EGC	90 days	Nuubo (textile wearable holter)	Accurate detection of paroxysmal atrial fibrillation compared to Holter.. o
[86]	Perez et al., 2023	USA	Prospective, pragmatic study	No	419297	N/R	Atrial fibrillation/ changes in blood flow, ECG	N/R	Apple watch, ECG patch	The ECG patch identified atrial fibrillation.
[87]	Rooney et al., 2019	USA	Observational, cross-sectional	No	2616	79	Atrial fibrillation/ ECG	2 weeks vs 4 weeks	Zio XT Patch	78% more subclinical atrial fibrillation was detected by 4 weeks of monitoring vs 2 weeks of ECG monitoring.
[88]	Schreiber et al., 2014	USA	Prospective, observational	No	174	52	Cardiac arrhythmia/ ECG	2 weeks	Zio Patch	Diagnostic yield for cardiac arrhythmias with the wearable device was 63.2%.
[89]	Steinhubl et al., 2018	USA	Observational, cohort	Yes	15214	72	Atrial fibrillation/ ECG	2-4 weeks	iRhythm ZioXT, skin adhesive patch	Atrial fibrillation was identified more in monitored groups than unmonitored. Monitoring was associated with increased initiation of anticoagulants, outpatient cardiology visits, and primary care visits, but no difference in emergency departments and hospitalizations.
[90]	Tison et al., 2018	USA	Cohort	No	1617	N/R	Arrhythmia/ heart rate, step count	N/R	Apple watch	The wearable detected atrial fibrillation against the reference standard 12-lead ECG.
[91]	Wouters et al., 2022	Belgium	Prospective RCT	Yes	40	69*	Cryptogenic stroke or transient ischemic attack/ photoplethysmography	180 days	Smartwatch	Device detected atrial fibrillation and other arrhythmias that were confirmed in some participants.
**Respiratory diseases**										
[92]	Mishra et al., 2020	USA	Prospective	Yes	5262 (32)	44	COVID-19/ heart rate, number of daily steps, time asleep	N/R	Smartwatches (Fitbits, Apple watches, Garmin, other)	Device detected COVID-19 before or at symptom onset.
[93]	Smarr et al., 2020	USA, UK, Finland, Austria, Canada, Germany, Honduras, Italy, Netherlands, Norway, Sweden	Feasibility	No	50	44	COVID-19/ skin temperature	65 days	Oura ring	Device detected temperature increases that correlated with self-reported fever. Wearable data showed that COVID-19 onset could be detected before first symptoms.
[94]	Radin et al., 2020	USA	Population-based	No	47249	43	Influenza-like illness/ mean resting heart rate, mean sleep time	N/R	Fitbit	Fitbit data significantly improved influenza-like illness predictions.

*age is for the experimental group only

Most of the included studies monitored changes in an existing disease (68 studies, 85%), while the remainder detected new diseases or risk factors for disease (12 studies, 15%). Of the 12 studies that identified new diseases, the majority were on cardiovascular (9/12, 75%), and the remainder were respiratory (3/12, 25%). Of the 68 studies that monitored an existing disease, the most common disease category was neurodegenerative disorders (22/68, 32%), followed by respiratory diseases (9/68, 13%), psychiatric disorders (9/68, 13%) and cardiac diseases and stroke (9/68, 13%). Other categories for existing disease were diabetes, cancer, musculoskeletal disorders, and renal disease.

### Wearable devices

**Fig 2:**
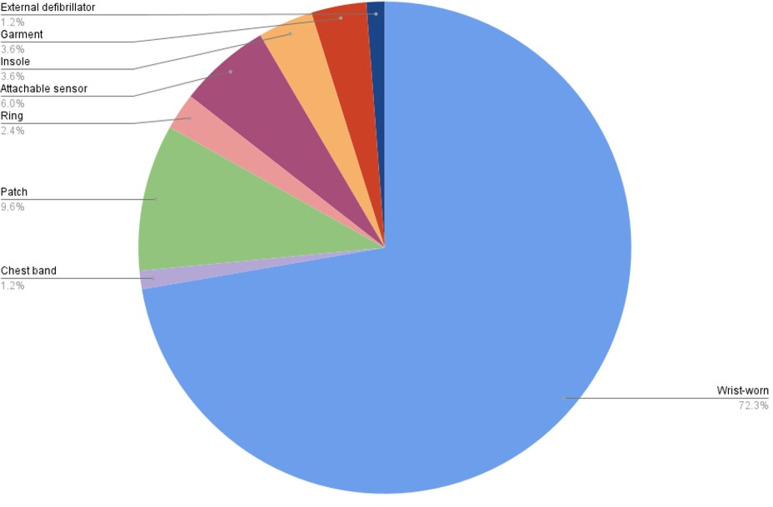
Device types. Light blue indicates wrist-worn devices (72.3% of total devices), purple indicates chest band (1.2%), green indicates patch devices (9.6%), light pink indicates ring wearables (2.4%), dark pink indicates attachable devices (6.0%), yellow indicates insole devices (3.6%), red indicates garment devices (3.6%), and dark blue indicates wearable defibrillator (1,2%).

Table 2 lists wearable device names and monitoring lengths for each of the included studies. Fig 2 and Table 3 show device types and brands. S2 File contains detailed information on device name, model, company, and sensor type for each study, organized by wearable device type. Studies used many different wearable device types, models, brands, and sensors. Some studies did not include details on device name (6/80, 8%), model (38/80, 48%), brand (11/80, 14%), sensor type (24/80, 30%), or body part (14/80, 18%). The most common device types were watches and bracelets (50 studies, 63%), and the most frequently used brand was Fitbit (22 studies, 42%). A wide variety of parameters were measured across studies, including movement or activity (37 studies, 46%), step count (33 studies, 41%), heart rate (15 studies, 19%), ECG (10 studies, 13%), sleep-related parameters (10 studies, 13%), temperature (7 studies, 9%), breathing rate (3 studies, 4%), blood oxygen saturation (3 studies, 4%), and blood pressure (2 studies, 3%).

**Table 3:** Device types, models and brands used in the included studies.

Device type	Device brand and model	Used in # of studies (%)
**Wrist-worn**	Wrist-worn, not specified	11 (14%)
	Senseband	2 (3%)
	Apple Watch Series 2	3 (4%)
	Apple Watch Series 4	1 (1%)
	FitBit Charge HR	3 (4%)
	FitBit Charge 2	3 (4%)
	FitBit Charge 3	3 (4%)
	FitBit Flex	5 (6%)
	FitBit - unspecified	4 (5%)
	FitBit Inspire 3	1 (1%)
	Garmin Vivofit 2	1 (1%)
	Gramin Vivofit	3 (4%)
	Garmin Vivosmart 4	1 (1%)
	Garmin - unspecified	1 (1%)
	Fitbit Alta	1 (1%)
	SweetAge	1 (1%)
	FitBit Versa	2 (3%)
	Mi Band 2 smartwatch	1 (1%)
	Actigraph GT9X Link	2 (3%)
	Verily Study Watch 2nd Generation	1 (1%)
	Personal KinetiGraph system	2 (3%)
	TriTrac RT3 accelerometer	1 (1%)
	PRO-Diary	1 (1%)
	Opal inertial sensors	1 (1%)
	Axixity AX3 triaxial accelerometer	1 (1%)
	Empatica E4	2 (3%)
	GENE Activ accelerometer	1 (1%)
	Philips Actiwatch	1 (1%)
**Chest band**	Chest band	1 (1%)
**Patch**	ZIO	5 (6%)
	Vital Connect	1 (1%)
	BioStamp	1 (1%)
	iRhythm ZioXT	1 (1%)
**Ring**	Oura ring	2 (3%)
**Attachable sensor**	MTx-W sensor	1 (1%)
	Cosinuss One in-ear sensor	1 (1%)
	STAT-ONTM system	1 (1%)
	DynaPort Hybrid system	1 (1%)
	SEEQ system	1 (1%)
**Insole**	SurroSense Rx	2 (3%)
	BioTrainer Pro	1 (1%)
**Garment**	Siren Diabetic Socks	1 (1%)
	Equivital EQ02 + LifeMonitor	1 (1%)
	Nuubo	1 (1%)
**External Defibrillator**	LifeVest	1 (1%)

Table 4 contains information on study methods related to wearable devices. Almost half of the included studies monitored participants for up to one month (33 studies, 41%). Seventeen studies (21%) monitored participants for one to three months, and 12 studies (15%) monitored participants for three to six months. Eight studies (10%) monitored participants for six months to one year. One study (1%) monitored patients for over one year. Forty-one of the included studies (51%) reported the onboarding instructions provided to participants, 25 studies (31%) included information on the frequency of device charging or removal from the body, and 13 studies (16%) included information on technical support with maintenance and setup of the device that was provided to study participants. One study (1%) used a device that required a prescription.

**Table 4:** Proportion of studies that reported details on monitoring protocols, compliance, useability, and technology literacy.

	Proportion of included studies
**Methods**	
Instructions on device use	n = 41, 51%
Frequency of charging or removal from the body	n = 25, 31%
Technical support with maintenance/setup	n = 13, 16%
**Results**	
Compliance (device wear time, study dropout)	n = 51, 64%
Patient reported useability	n = 13, 16%
Technology literacy data	n = 1, 1%

### Findings of the included studies

A summary of outcome-related findings for each of the 80 included studies can be found in Table 1. Six of the studies (9%) were randomized controlled trials (RCTs), and four of these RCTs (4/6, 67%) showed a positive clinical impact, including evidence of improved patient outcomes resulting from device use. Observational studies used a wide range of wearable devices and health parameters for a diverse set of diseases. All but two observational studies (62/64, 97%) found at least one significant correlation between a device parameter and a clinical outcome of interest. Table 3 shows studies that reported findings related to compliance, useability, and participant technology literacy. Over half of the included studies (51 studies, 64%) reported study protocol compliance, including device wear time or study dropout. Patient-reported usability was reported in 13 studies (16%). Only one study (1%) assessed technology literacy among the participants.

## Discussion

Remote health monitoring has the potential to improve healthcare significantly by enabeling continuous monitoring of fidrsdr pstsmryrtd in non-hospital settings, however, real-world impacts remain unproven. (8)(9)(10) In this review, we examined the current evidence for wearables in the medical realm, focusing on measurable effects on clinical outcomes. Building on prior work, we included a wide variety of disease contexts in non-hospital settings and a broadly inclusive set of devices.

The most commonly used devices in this review were watches and bracelets (50/80, 63%), with Fitbit Inc. being the most common brand (22/50, 42%). Wrist-worn devices offer key advantages in clinical settings due to their unobtrusive, comfortable design – similar to traditional wristwatches – which facilitates continuous wear during sleep and physical activity (3). This enables uninterrupted data collection and the potential for early detection of health anomalies [3]. Interestingly, some studies (6/80, 8%) also neglected to report the specific device model/brand using generic terms like “smartwatch”, “inclinometer”, or “wearable activity tracker”. Additional missing details included device model (38/80, 48%), manufacturer (11/80, 14%), sensor type (24/80, 30%), and body placement (14/80, 18%) (S2). Insufficient reporting of technical specifications limits cross-study comparisons and synthesis of findings.

Most included studies monitored an existing disease (68/80, 85%), while a smaller proportion aimed to diagnose a new disease (12/80, 15%). A wide variety of health conditions were monitored, including neurodegenerative disorders (22/68, 32%), respiratory disorders (9/68, 13%), psychiatric disorders (9/68, 13%), cardiac diseases and stroke (9/68, 13%), cancer (7/68, 10%) musculoskeletal disorders (6/68, 9%), diabetes (4/68, 6%), and renal disease (2/68, 3%). Among the studies targeting new disease diagnosis (12/80, 15%), two main categories emerged: cardiac diseases (9/12, 75%) and respiratory diseases (3/12, 25%).

Of the 80 studies included in this review, only six (6/80, 8%) were RCTs, focusing on diabetes (1/6, 17%) respiratory disorders (3/6, 50%), and neurodegenerative disorders (2/6, 33%). Four of these trials (4/6, 67%) showed a positive clinical impact, including evidence of improved patient outcomes associated with device use. Abbott et al (2019) demonstrated a 71% reduction in diabetic ulcer incidence with the use of SurroSense Rx, a smart insole system was used [25]. In COPD, Pedone et al (2013) reported a lower incidence of respiratory events in an experimental group monitored using a wristband tracking heart rate, physical activity, temperature and galvanic skin response, in combination with a commercial pulse-oximeter [40]. In multiple sclerosis (MS), Block et al (2021) found improved adherence to therapy recommendations in patients using a Fitbit Flex 2 but no improvements in disease symptoms [55]. Orme et al (2018) reported mixed effects for COPD using an inclinometer: no change in respiratory symptoms, but reduced fatigue levels [39]. Finally, Tabak et al (2014) found no difference in health status between COPD patients who used a belt-worn Mtx-W sensor and those who did not [42].

As this is a scoping review, we did not perform a systematic quality assessment of studies in this review, however, it is clear that the overall quality of evidence in included studies was low as there were few RCTs. Our findings on minimally published RCTs on this topic do however align with a 2018 meta-analysis reporting a lack of high-quality data on effectiveness for RHM and wearables [95]. In part, the paucity of high-quality data in this area can possibly be explained factors such as high cost associated with complex study protocols, significant implementation challenges, the need for industry partnerships, and complex ethical and legal barriers. There may also be a lack of buy-in from key stakeholders, including patients and doctors [96–98]. Given the paucity of evidence, the use of wearables and real-time data feedback for medical interventions, particularly in acute care, represents an emerging and largely uncharted frontier.

As mentioned, a large proportion of the reviewed publications used common, consumer-grade, wrist-worn devices, for example, fitness trackers or smartwatches. Despite concerns about their accuracy, commercial-grade devices have gained widespread popularity in the general population and therefore offer great potential for wide-spread health impacts. Save for a few select applications (ex. atrial fibrillation detection), these tools are marketed for non-medical use and lifestyle enhancement; however, the integration of their data in medical decision-making seems inevitable. Future research should attempt to test the medical utility of commercial-grade devices in the real-world.

Finally, a key factor in the successful implementation of RHM is patient interest and satisfaction [98]. Involving end users is essential to support patient-centered design principles that promote long-term adoption and usability [99,100]. However, most studies (41/80, 51%) did not report whether participants received device usage instructions. Proper education is critical, as improper use and motion artifacts can compromise data quality and analysis [101]. Additionally, only 13 studies (16%) described providing technical support for device setup or maintenance. This lack of support may contribute to the low compliance rates frequently observed in RHM studies [102,103]. Despite the importance of adherence, only 64% of studies in our review reported on participant compliance. Furthermore, just 16% included patient-reported data on device usability, and only 1% assessed baseline technology literacy. The absence of such data limits the ability to refine digital health interventions and adapt them to diverse populations. Future research should address not only the selection of wearable technologies, but also the critical role of patient education, ongoing technical support, and user engagement to enable successful and scalable implementation [104].

Another issue not well-addressed by the reviewed studies were concerns with data privacy and security. As artificial intelligence advances and risks related to data breaches and cyberattacks grow, the continuous transmission of data via wearables may raise significant privacy issues for patients. Future research must obtain appropriate ethical clearances and adhere to institutional and federal regulations, which may require the development of new policies governing continuous health monitoring technologies. In parallel, researchers and clinical innovators must prioritize the development of secure algorithms and communication channels to safeguard patient data. Compromised security could present a critical barrier to adoption and trust, particularly within research contexts. As wearable technologies become more prevalent in healthcare, careful consideration of regulatory frameworks and ethical requirements is essential to ensure patient safety and data protection.

## Limitations

There are several limitations inherent to this review. Since the field of RHM continues to develop and expand, there is a general lack of consensus around terminology, for example, definitions of ‘remote monitoring’ and ‘wearable device’ [105]. This posed a significant challenge when devising a search strategy and we likely did not capture all relevant studies. However, our eligibility criteria were broad compared to prior work [3,106,107], allowing us to capture studies on a wide range of devices and disorders. We also performed citation searching to identify studies that were missed by our search strategy. Importantly, we did not eliminate studies based on methodological quality and we included both consumer-grade and medical-grade technologies. We focused on wearable (not implantable) devices and our findings may not generalize to more invasive forms of RHM, including blood glucose monitors and pacemakers. We also did not include smartphones in our definition of wearable devices, although these can in theory be ‘worn’ if strapped to the body. Lastly, the fast pace of innovation complicates the generalizability of data on older devices included in our review.

## Conclusions

To our knowledge, our review is the most comprehensive to date for evidence of wearables influencing clinical outcomes in non-hospital settings. Our findings underscore the challenges of conducting timely and comprehensive evidence synthesis in this rapidly evolving field. The adoption of standardized terminology and consistent reporting on usability, acceptability, adherence, patient education, and technical support would enhance data quality and facilitate comparability across studies. High-quality RCT data is critically needed to establish the clinical utility of wearable technologies. Future research should be strategically prioritized to focus on technologies and applications most relevant to clinical practice and regulatory decision-making. Additionally, continued attention to patient privacy and data security will be essential to prevent unintended harms as these technologies are integrated into clinical care.

## Supporting information

S1 File Detailed version of the MEDLINE search strategy used in this scoping review. This search strategy was translated for use in other databases.
(DOCX)

S2 File Detailed information on the wearable devices used in each of the 59 included studies. (DOCX)
