# Supplementary material for: Wearables research for continuous monitoring of patient outcomes: A scoping review
Source: PLOS Digit Health. 2025 May 9;4(5):e0000860. doi: 10.1371/journal.pdig.0000860 (PMC12063813; doi:10.1371/journal.pdig.0000860)
Supplement: S1 File — This search strategy was translated for use in other databases. (DOCX) [file pdig.0000860.s001.docx]

**S1 File.** Detailed version of the MEDLINE search strategy used in this scoping review. This search strategy was translated for use in other databases.

**Ovid MEDLINE(R) ALL <1946 to February 22, 2023>**

1 (FitBit* or "Apple Watch*" or (Garmin and (Vivosmart* or vivofit* or vivoactive or vivomove or marq or forerunner or fenix or epix or venu or lily or instinct or enduro or quatix)) or (Samsung and (gear or galaxy-watch*)) or Polar-H7 or Polar-H10 or Polar-H9 or Polar-Ignite or polar-verity or Polar-Pacer or polar-vantage or polar-unite or polar-grit or "Microsoft Band" or "microsoft bands" or "mi smart band*" or "mi band" or "mi bands" or (xiaomi and (wristband or band or bands)) or (Zephyr and (BioHarness or bio-harness or biosensor or wearable or performance-systems or medtronic)) or pulsense or pulsesense or striiv or Hexoskin* or hexo-skin* or omshirt* or "om shirt*" or omgarment* or "om garment*" or "om bra*" or (ombra and (wearable or sensor or biosensor or ecg)) or OmSignal or "oura ring*").mp. 1673

2 (VitalPatch or VitalConnect or "vital connect" or "Accurate 24" or "BodyGuardian" or "VinCense" or Sensium or Zensor or ((SpryHealth or Spry-Health) and Loop) or (SEEQ and ("mobile cardiac telemetry" or MCT)) or vyvo or helo-watch or helo-vista or helo-sense or helo-icon or helo-extense or helo-lx or (Nuvant and ("mobile cardiac telemetry" or MCT)) or Kenzen or VisiMobile or "visi mobile" or Nuubo or QardioCore or equivital or lifemonitor or eqwave or Empatica or Everion or Snap40 or (Spire and health-tag) or biostrap).mp. 185

3 (fitness-tracker* or activity-tracker* or accelerometer* or accelerometre* or "smart watch*" or "smartwatch*" or "smart clothing" or "smart ring*" or "smart clothes" or "smart shirt*" or "smart band*" or "smart ring" or "smart rings" or Consumer wearable* or body sensor* or ((device* or sensor* or monitor or monitors or telemonitor or biosensor* or smart) adj5 (wearable or chest strap* or wristband* or wrist band* or wrist strap* or ankle strap* or clothing or clothes or shirt or shirts or blouse or blouses or shorts or pants or trousers or belt or belts or necklace* or bracelet* or anklet* or non-invasive or noninvasive or patch or patches or arm band* or armband* or cutaneous or transdermal or skin))).mp. 51467

4 ((ambulatory adj4 monitor*) and (wearable or chest strap* or wristband* or wrist band* or wrist strap* or ankle strap* or clothing or clothes or shirt or shirts or blouse or blouses or shorts or pants or trousers or belt or belts or necklace* or bracelet* or anklet* or non-invasive or noninvasive or patch or patches or arm band* or armband* or cutaneous or transdermal)).mp. 3056

5 1 or 2 or 3 or 4 53620

6 vital signs/ or blood pressure/ or body temperature/ or heart rate/ or respiratory rate/ or Oxygen Saturation/ 437417

7 outpatients/ 20776

8 (patients or vitals or vital-sign* or vital-function* or vital-parameter* or body-temperature or pulse-rate or respiration-rate or oxygen-saturation or SaO2 or O2Sat or SvO2 or blood-oxygen or heart-rate or blood-pressure or respiratory-rate or health metrics or recovery metrics or physiological parameters or physiological data or physiological signs or clinical parameters or clinical data or clinical signs or health status).mp. 7654531

9 (Outpatient* or ambulatory or telemetry or Telehealth or tele-health or Telemedicine or tele-medicine or telerehab* or tele-rehab* or digital-health or mobile-health or m-health or e-health or mhealth or ehealth or wireless-health).mp. 436118

10 6 or 7 or 8 or 9 7821618

11 (monitor* or surveillance or remot* measur* or telemetry).mp. 1409457

12 Monitoring, Physiologic/ 58509

13 monitoring, ambulatory/ or blood pressure monitoring, ambulatory/ or electrocardiography, ambulatory/ 31366

14 (physiologic* adj4 monitor*).mp. 62160

15 (telemonitor* or tele-monitor* or home monitor* or ambulatory monitor* or patient monitor* or monitor* patient*).mp. 19435

16 (10 and 11) or 12 or 13 or 14 or 15 568890

17 5 and 16 14069

18 (outpatient* or ambulatory or remote* or postop* or telemetry or Telehealth or tele-health or Telemedicine or tele-medicine or telerehab* or tele-rehab* or telemonitor* or tele-monitor* or digital-health or mobile-health or m-health or e-health or mhealth or ehealth or wireless-health or transtelephon* or home or house or daily life or daily lives or smartphone* or smart phone*).mp. 1848148

19 17 and 18 8021

20 ((continuous* adj6 (monitor* or data or analytic* or information or parameters)) or real-time).mp. 417578

21 19 and 20 1778

22 (Hospitalized or hospitalised or inpatient or inpatients or ward or wards or icu-patient* or "critical care" or "acute care").ti. 96007

23 21 not 22 1757

24 (((physical-activity or walking or step or steps or stepping or exercise) adj3 (promot* or increase or intervention* or levels or program or programs)) or activity-level* or behaviour*-change or behavior*-change or behaviour*-intervention* or behavior*-intervention* or healthy-lifestyle* or weight-reduction or weight loss or sedentary-behavior* or sedentary-behaviour*).ti. 47937

25 23 not 24 1740
