# Supplementary material for: Wearables research for continuous monitoring of patient outcomes: A scoping review
Source: PLOS Digit Health. 2025 May 9;4(5):e0000860. doi: 10.1371/journal.pdig.0000860 (PMC12063813; doi:10.1371/journal.pdig.0000860)
Supplement: S2 File — (DOCX) [file pdig.0000860.s002.docx]

**S2 File.** Detailed information on the wearable devices used in each of the 59 included studies.

| **Study** | | **Wearable device name** | **Wearable device model** | **Company** | **Sensor type** | **Body part** |
| --- | --- | --- | --- | --- | --- | --- |
| **Watches and bracelets** | | | | | |  |
| [1] | Alinia et al., 2021 | Empatica E4 wristband | E4 | Empatica Inc. | Photoplethysmography, electrodermal activity, 3-axis accelerometer, infrared thermopile | Wrist |
| [2] | Block et al., 2016 | Fitbit Flex | Flex | Fitbit Inc., San Francisco, CA, USA | Accelerometer | Wrist |
| [3] | Block et al., 2017 | Fitbit Flex 2 | Flex 2 | Fitbit Inc., San Francisco, CA, USA | Triaxial Accelerometer | Wrist |
| [4] | Block et al., 2019 | Fitbit Flex | Flex | Fitbit Inc. | Accelerometer | Wrist |
| [5] | Block et al., 2021 | Fitbit Flex 2 | Flex 2 | Fitbit | N/R | Wrist |
| [6] | Burq et al., 2021 | Verily study watch | Second generation | Verily | Triaxial accelerometer, gyroscope, inertial measurement unit, photoplethysmography, skin conductance sensors | Wrist |
| [7] | Carreiro et al., 2020 | E4 wearable sensor | N/R | Empatica, Milan, Italy | Three-axis accelerometer, electrodermal activity, skin temperature | Wrist |
| (56) |  |  |  |  |  |  |
| [8] | Cohen et al., 2022 | Garmin Vivofit 2 | Vivofit 2 | Garmin Ltd., Olathe, KS | Accelerometer | Arm |
| [9] | Dadhania et al., 2023 | Axivity AX3 | AX3 | Open Lab, Newcastle University | Triaxial accelerometer | Wrist |
| [10] | Dalla Costa et al., 2024 | Fitbit Charge 3 | Charge 3 | Fitbit | N/R | Dominant hand |
| [11] | Davies et al., 2020 | 3D accelerometer wrist-worn device | N/R | N/R | 3D accelerometer | Wrist |
| [12] | Drent et al., 2020 | Fitbit Charge HR | Charge HR | Fitbit Inc., San Francisco, CA, USA | N/R | Wrist |
| [13] | Faye-Karmon et al., 2024 | Apple watch | Series 4, 40mm | Apple | Accelerometer and gyroscope | Wrist |
| [14] | Gresham et al., 2018 | Fitbit Charge HR | Charge HR | N/R | N/R | Wrist |
| [15] | Gordon et al., 2019 | Smartwatch | N/R | N/R | Triaxial accelerometer | Wrist |
| [16] | Joshi et al., 2019 | Personal KinetiGraph watch | N/R | Developed by neurologists at the Melbourne-based Florey Institute of Neuroscience and Mental Health | 3-axis iMEMS accelerometer (ADXL345 Analog Devices) | Wrist |
| [17] | Kim et al., 2020 | Bluetooth sphygmomanometer A&D UA-651BLE | A&D UA-651BLE | A&D Engineering, Inc, San Jose, California | Sphygmomanometer | Wrist |
|  |  | Wrist-worn smart band | N/R | Croise S, Patron Co, Ltd, Gyeonggi-do, Republic of Korea | N/R | Wrist |
| [18] | Knight et al., 2018 | Fitbit, Garmin | N/R | Fitbit, Garmin | N/R | Wrist |
| [19] | Kolk et al., 2023 | GENEActiv accelerometer | N/R | Activinsights Ltd, Cambridgeshire, UK | Accelerometer | Wrist |
| [20] | Korde et al., 2023 | Garmin Vivofit | Vivofit | Garmin | N/R | N/R |
| [21] | Kotschet et al., 2023 | Parkinson’s KinetiGraph system | N/R | PKG, Global Kinetics Corporation, Australia | Accelerometers | Wrist |
| [22] | Kratz et al., 2019 | PRO-Diary | N/R | CamNTech, Cambridge, UK | Accelerometer | Wrist |
| [23] | Lahti et al., 2021 | Philips Actiwatch | N/R | Philips | Actigraphy system | Wrist |
|  |  | Garmin Vivofit wristband | N/R | Garmin | N/R | Wrist |
| [24] | Lee et al., 2022 | Fitbit Charge HR | Charge HR 2 or 3 | Fitbit Inc | N/R | Wrist |
| [25] | Lunney et al., 2021 | Fitbit Alta HR tracker | Alta | Fitbit Inc. | N/R | Upper extremity |
| [26] | Low et al., 2017 | Fitbit Charge HR | Charge HR | Fitbit | Accelerometer | Wrist |
| [27] | Low et al., 2021 | Fitbit Charge | Charge 2 | Fitbit | N/R | Wrist |
| [28] | Low et al., 2024 | Fitbit Inspire 3 | Inspire 3 | Fitbit | N/R | Wrist |
| [29] | Maharaj et al., 2022 | Mi Band 2 | 2 | N/R | Accelerometer | Wrist |
| [30] | Mahoney et al., 2023 | Garmin Vivosmart 4 | Vivosmart 4 | Garmin | Accelerometry, photoplethysmography | N/R |
| [31] | Mishra et al., 2020 | Smartwatches (Fitbits (Fitbit Ionic, Charge 4 and Charge 3), Apple watches, Garmin, other) | Fitbit Ionic, Charge 4, and Charge 3 | Fitbit, Apple, Garmin | N/R | Wrist |
| [32] | Motl et al., 2011 | ActiGraph | Model 7164 | Manufacturing Technology Incorporated, Fort Walton Beach, FL, USA | Single-axis accelerometer | N/R |
| [33] | O’Brien et al., 2017 | Wrist-worn device | N/R | N/R | Tri-axial accelerometer | Wrist |
| [34] | Ohri et al., 2019 | Garmin Vivofit | Vivofit | Garmin Vivofit, Lenexa, KS | N/R | N/R |
| (17) |  |  |  |  |  |  |
| [35] | Patterson et al., 2020 | Fitbit Flex | Flex | Fitbit  Corp, San Francisco, CA | N/R | Wrist |
| [36] | Pedone et al., 2023 | SweetAge monitoring system wristband | N/R | Intersistemi SpA, Rome, and Evolvo srl, Rome | Sensors for heart rate, physical activity, near-body temperature, and galvanic skin response | Wrist |
|  |  | SweetAge monitoring system pulse-oximeter | N/R | Nonin Medical Inc. | Pulse oximeter | Wrist |
| [37] | Perraudin et al., 2018 | Actigraph Gt9X Link | Gt9X Link | Actigraph | Accelerometer | Wrist |
| [38] | Perez et al., 2019 | Apple watch | N/R | Apple | Photoplethysomography | Wrist |
| [39] | Powers et al., 2021 | Apple watch series 2 and above | Series 2 and above | N/R | Accelerometer, gyroscope | Wrist |
| [40] | Radin et al., 2020 | Fitbit | N/R | N/R | N/R | N/R |
| [41] | Scheer et al., 2017 | Fitbit Flex | Flex | Fitbit Inc. | 3-dimensional accelerometer | Wrist |
| [42] | Sun et al., 2023 | Fitbit Charge 2 or 3 | Charge 2 or 3 | Fitbit | N/R | Wrist |
| [43] | Stollfuss et al., 2021 | Apple watch | Series 2 | Apple | Motion sensors | Wrist |
| [44] | Tison et al., 2018 | Apple watch | N/R | Apple Inc. | Photoplethysmography | - |
| [45] | Van Vugt et al., 2001 | Wrist-worn activity monitor | N/R | Gaehwiler Electronic, Switzerland | Accelerometer | Wrist |
| [46] | Woelfle et al., 2023 | Fitbit Versa 2 | Versa 2 | Fitbit | N/R | N/R |
| [47] | Wouters et al., 2022 | Smartwatch | N/R | N/R | Photoplethysmography | Wrist |
| [48] | Wu et al., 2021 | Fitbit Versa | Versa | Fitbit | N/R | Wrist |
| [49] | Yamagami et al., 2021 | Fitbit Charge 3 | Charge 3 | Fitbit Inc | N/R | Wrist |
| [50] | Zhang et al., 2021 | Fitbit Charge 2 or 3 | Charge 2 or 3 | Fitbit Inc | Photoplethysmography | Wrist |
| **Armbands** | | | | | | |
| [51] | Cereda et al., 2010 | SenseWear Armband | N/R | SenseWear | N/R | Triceps region |
| [52] | Okumus et al., 2018 | Sensewear armband | N/R | BodyMedia SenseWear armband, USA | Motion, steps, galvanic skin response, heat flux, skin temperature | N/R |
| **Belts** | | | | | |  |
| [53] | Armstrong et al., 2004 | Biotrainer Pro | N/R | IM Systems, Boston, MA | Accelerometer, pedometer | Waist-worn |
| [54] | Caballol et al., 2023 | STAT-ON wearable inertial sensor | N/R | STAT-ON | N/R | Worn on hip and wrist |
| [55] | Klassen et al., 2008 | TriTrac RT3 accelerometer | TriTrac RT3 | StayHealthy Inc., Monrovia, CA, USA | Tri-axial accelerometer | Worn on a belt |
| [56] | Mancini et al., 2015 | Opal inertial sensors | N/R | APDM, Inc., Portland, OR, USA | Gyroscope | Worn on belt and each foot |
| [57] | Tabak et al., 2014 | Mtx-W sensor | N/R | Xsens Technologies, Enschede, The Netherlands | 3-D accelerometer | Worn on the subject’s belt |
| [58] | Shammas et al., 2014 | Move II activity sensor | Move 2 | Movisens GmbH, Karlsruhe, Germany | 3-axial accelerometer | One on the hip and two on the ankles |
| [59] | Weiss et al., 2014 | DynaPort Hybrid system | N/R | McRoberts, DynaPort Hybrid system, The Netherlands | Triaxial accelerometer | On a belt on the lower back |
| **Skin adhesive patches** | | | | | |  |
| [60] | Gurchiek et al., 2019 | BioStamp | N/R | MC10 Inc., Lexington, MA | Tri-axial accelerometer, surface electromyography | Muscle belly of rectus femoris and wrist |
| [61] | Ha et al., 2021 | SEEQ system  CardioSTAT system | N/R | Medtronic  Icentia | ECG | Anterior left chest |
| [38] | Perez et al., 2019 | ECG patch | N/R | N/R | ECG | N/R |
| [62] | Pothineni et al., 2022 | ZIO XT Patch | ZIO XT | iRhythm Technologies, San Francisco, CA | ECG | Chest |
| [63] | Rooney et al., 2019 | Zio XT Patch | ZIO XT | iRhythm Technologies, San Francisco, CA | ECG | N/R |
| [64] | Rosenberg et al., 2013 | Zio XT Patch | ZIO XT | iRhythm Technologies, San Francisco, CA, USA | ECG | N/R |
| [65] | Schreiber et al., 2014 | Zio Patch | ZIO | iRhythm Technologies, San Francisco, CA | ECG | Chest |
| [66] | Stehlik et al., 2020 | Wearable sensor patch | N/R | Vital Connect, San Jose, CA | 3-axis accelerometer, temperature sensor, ECG | Chest |
| [67] | Steinhubl et al., 2018 | iRhythm ZioXT Patch | ZioXT | iRhythm | ECG | N/R |
| [68] | Tung et al., 2014 | ZIO patch | ZIO | iRhythm Technologies, San Francisco, CA | ECG | N/R |
| **Rings** | | | | | |  |
| [69] | Moshe et al., 2021 | Oura ring | N/R | Oura | 3D accelerometer | Finger |
| [70] | Smarr et al., 2020 | Oura ring | N/R | Oura | Photoplethysmogram | Finger |
| **In-ear sensor** | | | | | | |
| [71] | Wurzer et al., 2021 | Cosinuss One in-ear sensor | Cosinuss One | Cosinuss GmbH, Munich, Germany | Photoplethysmogram | Ear |
| **Vest or chest strap** | | | | | | |
| [72] | Garcia et al., 2023 | LifeVest, external defibrillator | N/R | ZOLL Cardiac Management Solutions, PA | N/R | N/R |
| [73] | Hawthorne et al., 2022 | EQ02+ LifeMonitor | EQ02+ | Equivital, Cambridge, UK | N/R | Chest |
| [74] | Rubio et al., 2017 | Mounted accelerometer | N/R | N/R | Tri-axial accelerometer | Chest |
| **Clothing/textiles** | | | | | |  |
| [75] | Pagola et al., 2023 | Nuubo (textile wearable holter) | N/R | Nuubo | ECG | Chest |
| [76] | Reyzelman et al., 2018 | Siren Diabetic Socks | N/R | Neuro Fabric, Siren Care Inc, San Francisco, CA | Temperature monitoring sensors | Feet |
| **Insoles** | | | | | | |
| [77] | Abbott et al., 2019 | SurroSense Rx, smart insole system | N/R | SurroSense Rx, Orpyx Medical Technologies, Canada | Eight pressure sensors | Foot |
| [78] | Najafi et al., 2017 | The SurroSense Rx, smart insole system | N/R | The SurroSense Rx, Orpyx Medical Technologies Inc, Calgary, Canada | Eight pressure sensors | Foot |
| **Ankle-worn devices** | | | | | | |
| [79] | Toogood et al., 2016 | Fitbit | N/R | San Francisco, California | Accelerometer | Ankle |
| **Unclear** | | | | | |  |
| [80] | Orme et al., 2018 | Inclinometer | N/R | N/R | Inclinometer | Waist |

**References**

1. Alinia P, Sah RK, McDonell M, Pendry P, Parent S, Ghasemzadeh H, et al. Associations Between Physiological Signals Captured Using Wearable Sensors and Self-reported Outcomes Among Adults in Alcohol Use Disorder Recovery: Development and Usability Study. JMIR Form Res. 2021;5:e27891-.

2. Block VJ. Fitbit Remote-Monitoring in Multiple Sclerosis: FITriMS Study. Fitbit Remote-monitoring in Multiple Sclerosis: FITriMS Study. 2016;

3. Block VJ, Lizee A, Crabtree-Hartman E, Bevan CJ, Graves JS, Bove R, et al. Continuous daily assessment of multiple sclerosis disability using remote step count monitoring. J Neurol. 2017;264:316–26.

4. Block VJ, Bove R, Zhao C, Garcha P, Graves J, Romeo AR, et al. Association of Continuous Assessment of Step Count by Remote Monitoring With Disability Progression Among Adults With Multiple Sclerosis. JAMA Netw Open. 2019;2:e190570-.

5. Block VJ, Gopal A, Rowles W, -Yueh C, Gelfand JM, Bove R. CoachMS, an innovative closed-loop, interdisciplinary platform to monitor and proactively treat MS symptoms: A pilot study. Mult Scler J Exp Transl Clin. 2021;7:2055217321988937-.

6. Burq M, Rainaldi E, Ho KC, Chen C, Bloem BR, Evers LJW, et al. Virtual Exam for Parkinson’s Disease Enables Frequent and Reliable Remote Measurements of Motor Function. medRxiv [Internet]. 2021; Available from: https://www.medrxiv.org/

7. Carreiro S, Chintha KK, Shrestha S, Chapman B, Smelson D, Indic P. Wearable sensor-based detection of stress and craving in patients during treatment for substance use disorder: A mixed methods pilot study. Drug Alcohol Depend. 2020;209:107929.

8. Cohen B, Munugoti S, Kotwani S, Randhawa LS, Dalezman S, Elters AC, et al. Continuous Long-Term Physical Activity Monitoring in Hemodialysis Patients. Kidney360. 2022;3:1545–55.

9. Dadhania S, Pakzad-Shahabi L, Mistry S, Williams M. Triaxial accelerometer-measured physical activity and functional behaviours among people with High Grade Glioma: The BrainWear Study. PLoS One. 2023;18.

10. Dalla Costa G, Nos C, Zabalza A, Buron M, Magyari M, Sellebjerg F, et al. A wearable device perspective on the standard definitions of disability progression in multiple sclerosis. Multiple Sclerosis Journal. 2024;30.

11. Davies EH, Johnston J, Toro C, Tifft CJ. A feasibility study of mHealth and wearable technology in late onset GM2 gangliosidosis (Tay-Sachs and Sandhoff Disease). Orphanet J Rare Dis. 2020;15:199.

12. Drent M, Elfferich M, Breedveld E, J DV, Strookappe B. Benefit of wearing an activity tracker in sarcoidosis. J Pers Med [Internet]. 2020;10:1–11. Available from: https://www.mdpi.com/2075-4426/10/3/97/pdf

13. Fay-Karmon T, Galor N, Heimler B, Zilka A, Bartsch RP, Plotnik M, et al. Home-based monitoring of persons with advanced Parkinson’s disease using smartwatch-smartphone technology. Sci Rep. 2024;14.

14. Gresham G, Hendifar AE, Neeman E, Tuli R, Rimel BJ, Figlin RA, et al. Wearable activity monitors to assess performance status and predict clinical outcomes in advanced cancer patients. NPJ Digit Med [Internet]. 2018;1. Available from: https://login.ezproxy.library.ualberta.ca/login?url=https://search.ebscohost.com/login.aspx?direct=true&db=edselc&AN=edselc.2-52.0-85089606338&site=eds-live&scope=site

15. Gordon MF, Grachev ID, Mazeh I, Dolan Y, Reilmann R, Loupe PS, et al. Quantification of Motor Function in Huntington Disease Patients Using Wearable Sensor Devices. Digit Biomark. 2019;3:103–15.

16. Joshi R, Joshi M, Bronstein JM, Keener A, Yang DD, Alcazar J, et al. PKG Movement Recording System Use Shows Promise in Routine Clinical Care of Patients With Parkinson’s Disease. Front Neurol [Internet]. 2019;10. Available from: https://login.ezproxy.library.ualberta.ca/login?url=https://search.ebscohost.com/login.aspx?direct=true&db=edselc&AN=edselc.2-52.0-85073669187&site=eds-live&scope=site

17. Kim DY, Kwon H, K.-W. N, Lee Y, H.-M. K, Chung YS. Remote management of poststroke patients with a smartphone-based management system integrated in clinical care: Prospective, nonrandomized, interventional study. J Med Internet Res [Internet]. 2020;22:e15377-. Available from: https://www.jmir.org/2020/2/e15377/pdf

18. Knight A, Bidargaddi N. Commonly available activity tracker apps and wearables as a mental health outcome indicator: A prospective observational cohort study among young adults with psychological distress. J Affect Disord [Internet]. 2018;236:31–6. Available from: https://www.elsevier.com/locate/jad

19. Kolk MZH, Frodi DM, Langford J, Meskers CJ, Andersen TO, Jacobsen PK, et al. Behavioural digital biomarkers enable real-time monitoring of patient-reported outcomes: a substudy of the multicenter, prospective observational SafeHeart study. Eur Heart J Qual Care Clin Outcomes. 2023;

20. Korde N, Tavitian E, Mastey D, Lengfellner J, Hevroni G, Zarski A, et al. Association of patient activity bio-profiles with health-related quality of life in patients with newly diagnosed multiple myeloma: a prospective observational cohort study. EClinicalMedicine. 2023;57.

21. Kotschet K, Osborn S, Horne M. Measurement of bradykinesia and chorea in Huntington’s Disease using ambulatory monitoring. Clin Park Relat Disord. 2023;8:100179.

22. Kratz AL, Fritz NE, Braley TJ, Scott EL, Foxen-Craft E, Murphy SL. Daily Temporal Associations Between Physical Activity and Symptoms in Multiple Sclerosis. Ann Behav Med. 2019;53:98–108.

23. Lahti AC, Wang D, Pei H, Baker S, Narayan VA. Clinical Utility of Wearable Sensors and Patient-Reported Surveys in Patients With Schizophrenia: Noninterventional, Observational Study. JMIR Ment Health. 2021;8:e26234-.

24. Lee H-J, Cho C-H, Lee T, Jeong J, Yeom JW, Kim S, et al. Prediction of impending mood episode recurrence using real-time digital phenotypes in major depression and bipolar disorders in South Korea: a prospective nationwide cohort study. Psychol Med. 2022;1–9.

25. Lunney M, Kusi-Appiah E, Lewis R, Tonelli M, Ferber R, Wiebe N, et al. Wearable Fitness Trackers to Predict Clinical Deterioration in Maintenance Hemodialysis: A Prospective Cohort Feasibility Study. Kidney Med [Internet]. 2021;3:768-775.e1768. Available from: https://login.ezproxy.library.ualberta.ca/login?url=https://search.ebscohost.com/login.aspx?direct=true&db=edselc&AN=edselc.2-52.0-85111738351&site=eds-live&scope=site

26. Low CA, Dey AK, Ferreira D, Kamarck T, Sun W, Bae S, et al. Estimation of Symptom Severity During Chemotherapy From Passively Sensed Data: Exploratory Study. J Med Internet Res. 2017;19:e420-.

27. Low CA, Li M, Vega J, Durica KC, Ferreira D, Tam V, et al. Digital Biomarkers of Symptom Burden Self-Reported by Perioperative Patients Undergoing Pancreatic Surgery: Prospective Longitudinal Study. JMIR Cancer. 2021;7:e27975-.

28. Low CA, Bartel C, Fedor J, Durica KC, Marchetti G, Rosso AL, et al. Associations between performance-based and patient-reported physical functioning and real-world mobile sensor metrics in older cancer survivors: A pilot study. J Geriatr Oncol. 2024;15.

29. Maharaj M, Natarajan P, Fonseka RD, Khanna S, Choy WJ, Rooke K, et al. The concept of recovery kinetics: an observational study of continuous post-operative monitoring in spine surgery. J Spine Surg. 2022;8:196–203.

30. Mahoney JJ, Finomore VS, Marton JL, Ramadan J, Hodder SL, Thompson-Lake DGY, et al. Identifying biomarkers of drug use recurrence using wearable device technologies and phone applications. Drug Alcohol Depend. 2023;249.

31. Mishra T, Wang M, Metwally AA, Bogu GK, Brooks AW, Bahmani A, et al. Pre-symptomatic detection of COVID-19 from smartwatch data. Nat Biomed Eng [Internet]. 2020;4:1208–20. Available from: https://www.nature.com/natbiomedeng/

32. Motl RW, McAuley E. Association between change in physical activity and short-term disability progression in multiple sclerosis. J Rehabil Med [Internet]. 2011;43:305–310305. Available from: https://login.ezproxy.library.ualberta.ca/login?url=https://search.ebscohost.com/login.aspx?direct=true&db=edselc&AN=edselc.2-52.0-79953287028&site=eds-live&scope=site

33. O’Brien JT, Gallagher P, Stow D, Firbank M, Ferrier IN, Hammerla N, et al. A study of wrist-worn activity measurement as a potential real-world biomarker for late-life depression. Psychol Med [Internet]. 2017;47:93–10293. Available from: https://login.ezproxy.library.ualberta.ca/login?url=https://search.ebscohost.com/login.aspx?direct=true&db=edselc&AN=edselc.2-52.0-84988719249&site=eds-live&scope=site

34. Ohri N, Bodner WR, Kalnicki S, Garg M, Halmos B, Cheng H, et al. Daily Step Counts: A New Prognostic Factor in Locally Advanced Non-Small Cell Lung Cancer? Int J Radiat Oncol Biol Phys [Internet]. 2019;105:745–751745. Available from: https://login.ezproxy.library.ualberta.ca/login?url=https://search.ebscohost.com/login.aspx?direct=true&db=edselc&AN=edselc.2-52.0-85073550963&site=eds-live&scope=site

35. Patterson JT, Wu H-H, Chung CC, Bendich I, Barry JJ, Bini SA. Wearable activity sensors and early pain after total joint arthroplasty. Arthroplast Today. 2020;6:68–70.

36. Pedone C, Chiurco D, Scarlata S, Incalzi RA. Efficacy of multiparametric telemonitoring on respiratory outcomes in elderly people with COPD: a randomized controlled trial. BMC Health Serv Res [Internet]. 2013;13:1–7. Available from: https://login.ezproxy.library.ualberta.ca/login?url=https://search.ebscohost.com/login.aspx?direct=true&db=a9h&AN=88990196&site=eds-live&scope=site

37. Perraudin CGM, Illiano VP, Calvo F, O’Hare E, Donnelly SC, Mullan RH, et al. Observational Study of a Wearable Sensor and Smartphone Application Supporting Unsupervised Exercises to Assess Pain and Stiffness. Digit Biomark. 2018;2:106–25.

38. Perez M V., Mahaffey KW, Hedlin H, Rumsfeld JS, Garcia A, Ferris T, et al. Large-Scale Assessment of a Smartwatch to Identify Atrial Fibrillation. New England Journal of Medicine. 2019;381.

39. Powers R, Etezadi-Amoli M, Arnold EM, Kianian S, Mance I, Gibiansky M, et al. Smartwatch inertial sensors continuously monitor real-world motor fluctuations in Parkinson’s disease. Sci Transl Med. 2021;13.

40. Jennifer M Radin P, Nathan E Wineinger P, Eric J Topol P, Steven R Steinhubl MD. Harnessing wearable device data to improve state-level real-time surveillance of influenza-like illness in the USA: a population-based study. Lancet Digit Health [Internet]. 2020;2:e85–93. Available from: https://login.ezproxy.library.ualberta.ca/login?url=https://search.ebscohost.com/login.aspx?direct=true&db=edsdoj&AN=edsdoj.0b19ae2d635a4893af58fc85285cdef4&site=eds-live&scope=site

41. Scheer JK, Bakhsheshian J, Keefe MK, Lafage V, Bess S, Protopsaltis TS, et al. Initial Experience With Real-Time Continuous Physical Activity Monitoring in Patients Undergoing Spine Surgery. Clin Spine Surg. 2017;30:E1434–43.

42. Sun S, Folarin AA, Zhang Y, Cummins N, Garcia-Dias R, Stewart C, et al. Challenges in Using mHealth Data From Smartphones and Wearable Devices to Predict Depression Symptom Severity: Retrospective Analysis. J Med Internet Res. 2023;25.

43. Stollfuss B, Richter M, Dromann D, Klose H, Schwaiblmair M, Gruenig E, et al. Digital Tracking of Physical Activity, Heart Rate, and Inhalation Behavior in Patients With Pulmonary Arterial Hypertension Treated With Inhaled Iloprost: Observational Study (VENTASTEP). J Med Internet Res. 2021;23:e25163-.

44. Tison GH, Sanchez JM, Ballinger B, Singh A, Olgin JE, Pletcher MJ, et al. Passive Detection of Atrial Fibrillation Using a Commercially Available Smartwatch. JAMA Cardiol. 2018;3:409–16.

45. van Vugt JPP, Siesling S, Piet KKE, Middelkoop HAM, van Hilten JJ, Roos RAC, et al. Quantitative assessment of daytime motor activity provides a responsive measure of functional decline in patients with Huntington’s disease. Movement Disorders [Internet]. 2001;16:481–488481. Available from: https://login.ezproxy.library.ualberta.ca/login?url=https://search.ebscohost.com/login.aspx?direct=true&db=edselc&AN=edselc.2-52.0-0035353731&site=eds-live&scope=site

46. Woelfle T, Pless S, Reyes Ó, Wiencierz A, Kappos L, Granziera C, et al. Smartwatch-derived sleep and heart rate measures complement step counts in explaining established metrics of MS severity. Mult Scler Relat Disord. 2023;80.

47. Wouters F, Gruwez H, Vranken J, Vanhaen D, Daelman B, Ernon L, et al. The Potential and Limitations of Mobile Health and Insertable Cardiac Monitors in the Detection of Atrial Fibrillation in Cryptogenic Stroke Patients: Preliminary Results From the REMOTE Trial. Front Cardiovasc Med. 2022;9:848914.

48. Wu CT, Li GH, Huang CT, Cheng YC, Chen CH, Chien JY, et al. Acute exacerbation of a chronic obstructive pulmonary disease prediction system using wearable device data, machine learning, and deep learning: Development and cohort study. JMIR Mhealth Uhealth. 2021;9.

49. Yamagami K, Nomura A, Kometani M, Shimojima M, Sakata K, Usui S, et al. Early Detection of Symptom Exacerbation in Patients With SARS-CoV-2 Infection Using the Fitbit Charge 3 (DEXTERITY): Pilot Evaluation. JMIR Form Res. 2021;5:e30819-.

50. Zhang Y, Folarin AA, Sun S, Cummins N, Bendayan R, Ranjan Y, et al. Relationship Between Major Depression Symptom Severity and Sleep Collected Using a Wristband Wearable Device: Multicenter Longitudinal Observational Study. JMIR Mhealth Uhealth. 2021;9:e24604-.

51. Cereda E, Pezzoli G, Barichella M. Role of an electronic armband in motor function monitoring in patients with Parkinson’s disease. Nutrition. 2010;26:240–2.

52. Okumus G, Aslan GK, Arseven O, Ongen G, Issever H, Kiyan E. The role of an activity monitor in the objective evaluation of patients with pulmonary hypertension. Clinical Respiratory Journal [Internet]. 2018;12:119–25. Available from: https://login.ezproxy.library.ualberta.ca/login?url=https://search.ebscohost.com/login.aspx?direct=true&db=a9h&AN=127287201&site=eds-live&scope=site

53. Armstrong DG, Lavery LA, Holtz-Neiderer K, Mohler MJ, Wendel CS, Nixon BP, et al. Variability in Activity May Precede Diabetic Foot Ulceration. Diabetes Care [Internet]. 2004;27:1980–4. Available from: https://login.ezproxy.library.ualberta.ca/login?url=https://search.ebscohost.com/login.aspx?direct=true&db=edsovi&AN=edsovi.00003458.200408000.00020&site=eds-live&scope=site

54. Caballol N, Bayes A, Prats A, Martin-Baranera M, Quispe P. Feasibility of a wearable inertial sensor to assess motor complications and treatment in Parkinson’s disease. PLoS One. 2023;18:e0279910-.

55. Klassen L, Schachter C, Scudds R. An exploratory study of two measures of free-living physical activity for people with multiple sclerosis. Clin Rehabil [Internet]. 2008;22:260–71. Available from: https://login.ezproxy.library.ualberta.ca/login?url=https://search.ebscohost.com/login.aspx?direct=true&db=edsovi&AN=edsovi.00012200.200803000.00008&site=eds-live&scope=site

56. Mancini M, El-Gohary M, Pearson S, McNames J, Schlueter H, Nutt JG, et al. Continuous monitoring of turning in Parkinson’s disease: Rehabilitation potential. NeuroRehabilitation. 2015;37:3–10.

57. Tabak M, Vollenbroek-Hutten MM, van der Valk PD, van der Palen J, Hermens HJ. A telerehabilitation intervention for patients with Chronic Obstructive Pulmonary Disease: a randomized controlled pilot trial. Clin Rehabil. 2014;28:582–91.

58. Shammas L, Zentek T, von Haaren B, Schlesinger S, Hey S, Rashid A. Home-based system for physical activity monitoring in patients with multiple sclerosis (Pilot study). Biomed Eng Online [Internet]. 2014;13:1–25. Available from: https://login.ezproxy.library.ualberta.ca/login?url=https://search.ebscohost.com/login.aspx?direct=true&db=a9h&AN=94465749&site=eds-live&scope=site

59. Weiss A, Herman T, Giladi N, Hausdorff JM. Objective Assessment of Fall Risk in Parkinson’s Disease Using a Body-Fixed Sensor Worn for 3 Days. 2014; Available from: https://login.ezproxy.library.ualberta.ca/login?url=https://search.ebscohost.com/login.aspx?direct=true&db=edshld&AN=edshld.1.12407046&site=eds-live&scope=site

60. Gurchiek RD, Choquette RH, Beynnon BD, Slauterbeck JR, Tourville TW, Toth MJ, et al. Open-Source Remote Gait Analysis: A Post-Surgery Patient Monitoring Application. Sci Rep. 2019;9:17966.

61. Ha ACT, Verma S, Mazer CD, Quan A, Yanagawa B, Latter DA, et al. Effect of Continuous Electrocardiogram Monitoring on Detection of Undiagnosed Atrial Fibrillation After Hospitalization for Cardiac Surgery: A Randomized Clinical Trial. JAMA Netw Open. 2021;4:e2121867-.

62. Pothineni NVK, Soliman EZ, Cushman M, Howard G, Howard VJ, Kasner SE, et al. Continuous cardiac rhythm monitoring post-stroke: A feasibility study in REGARDS. J Stroke Cerebrovasc Dis. 2022;31:106662.

63. Rooney MR, Soliman EZ, Lutsey PL, Norby FL, Loehr LR, Mosley TH, et al. Prevalence and Characteristics of Subclinical Atrial Fibrillation in a Community-Dwelling Elderly Population: The ARIC Study. Circ Arrhythm Electrophysiol. 2019;12:e007390-.

64. Rosenberg MA, Samuel M, Thosani A, Zimetbaum PJ. Use of a noninvasive continuous monitoring device in the management of atrial fibrillation: a pilot study. Pacing Clin Electrophysiol. 2013;36:328–33.

65. Schreiber D, Sattar A, Drigalla D, Higgins S. Ambulatory cardiac monitoring for discharged emergency department patients with possible cardiac arrhythmias. West J Emerg Med. 2014;15:194–8.

66. Stehlik J, Schmalfuss C, Bozkurt B, Nativi-Nicolau J, Wohlfahrt P, Wegerich S, et al. Continuous Wearable Monitoring Analytics Predict Heart Failure Hospitalization: The LINK-HF Multicenter Study. Circ Heart Fail. 2020;13.

67. Steinhubl SR, Waalen J, Edwards AM, Ariniello LM, Mehta RR, Ebner GS, et al. Effect of a Home-Based Wearable Continuous ECG Monitoring Patch on Detection of Undiagnosed Atrial Fibrillation: The mSToPS Randomized Clinical Trial. JAMA. 2018;320:146–55.

68. Tung CE, Su D, Turakhia MP, Lansberg MG. Diagnostic Yield of Extended Cardiac Patch Monitoring in Patients with Stroke or TIA. Front Neurol. 2014;5:266.

69. Moshe I, Terhorst Y, Opoku Asare K, Sander LB, Ferreira D, Baumeister H, et al. Predicting Symptoms of Depression and Anxiety Using Smartphone and Wearable Data. Front Psychiatry. 2021;12:625247.

70. Smarr BL, Aschbacher K, Fisher SM, Chowdhary A, Dilchert S, Puldon K, et al. Feasibility of continuous fever monitoring using wearable devices. Sci Rep. 2020;10:21640.

71. Wurzer D, Spielhagen P, Siegmann A, Gercekcioglu A, Gorgass J, Henze S, et al. Remote monitoring of COVID-19 positive high-risk patients in domestic isolation: A feasibility study. PLoS One. 2021;16:e0257095-.

72. Garcia R, Warming PE, Narayanan K, Defaye P, Guedon-Moreau L, Blangy H, et al. Dynamic changes in nocturnal heart rate predict short-term cardiovascular events in patients using the wearable cardioverter-defibrillator: from the WEARIT-France cohort study. Europace. 2023;25.

73. Hawthorne G, Richardson M, Greening NJ, Esliger D, Briggs-Price S, Chaplin EJ, et al. A proof of concept for continuous, non-invasive, free-living vital signs monitoring to predict readmission following an acute exacerbation of COPD: a prospective cohort study. Respir Res. 2022;23:102.

74. Rubio N, Parker RA, Drost EM, Pinnock H, Weir CJ, Hanley J, et al. Home monitoring of breathing rate in people with chronic obstructive pulmonary disease: observational study of feasibility, acceptability, and change after exacerbation. International Journal of COPD [Internet]. 2017;ume 12:1221–31. Available from: https://login.ezproxy.library.ualberta.ca/login?url=https://search.ebscohost.com/login.aspx?direct=true&db=edsdoj&AN=edsdoj.4d7c31f570a4dd3904169a08b8978ed&site=eds-live&scope=site

75. Pagola J, Juega J, Francisco-Pascual J, Rodriguez M, Dorado L, Martinez R, et al. Intensive 90-day textile wearable Holter monitoring: an alternative to detect paroxysmal atrial fibrillation in selected patients with cryptogenic stroke. Heart Vessels. 2023;38:114–21.

76. Reyzelman AM, Koelewyn K, Murphy M, Shen X, Yu E, Pillai R, et al. Continuous Temperature-Monitoring Socks for Home Use in Patients With Diabetes: Observational Study. J Med Internet Res. 2018;20:e12460-.

77. Abbott CA, Chatwin KE, Foden P, Hasan AN, Sange C, Rajbhandari SM, et al. Innovative intelligent insole system reduces diabetic foot ulcer recurrence at plantar sites: a prospective, randomised, proof-of-concept study. Lancet Digit Health. 2019;1:e308–18.

78. Najafi B, Ron E, Enriquez A, Marin I, Razjouyan J, Armstrong DG. Smarter Sole Survival: Will Neuropathic Patients at High Risk for Ulceration Use a Smart Insole-Based Foot Protection System? J Diabetes Sci Technol [Internet]. 2017;11:702–13. Available from: https://login.ezproxy.library.ualberta.ca/login?url=https://search.ebscohost.com/login.aspx?direct=true&db=edsovi&AN=edsovi.01410380.201711040.00008&site=eds-live&scope=site

79. Toogood PA, Abdel MP, Spear JA, Cook SM, Cook DJ, Taunton MJ. The monitoring of activity at home after total hip arthroplasty. Bone and Joint Journal [Internet]. 2016;98-B:1450–14541450. Available from: https://login.ezproxy.library.ualberta.ca/login?url=https://search.ebscohost.com/login.aspx?direct=true&db=edselc&AN=edselc.2-52.0-85019058954&site=eds-live&scope=site

80. Orme MW, Weedon AE, Saukko PM, Esliger DW, Morgan MD, Steiner MC, et al. Findings of the Chronic Obstructive Pulmonary Disease-Sitting and Exacerbations Trial (COPD-SEAT) in Reducing Sedentary Time Using Wearable and Mobile Technologies With Educational Support: Randomized Controlled Feasibility Trial. JMIR Mhealth Uhealth. 2018;6:e84-.
